# Oxidative Catalytic Depolymerization of Lignin into Value-Added Monophenols by Carbon Nanotube-Supported Cu-Based Catalysts

**DOI:** 10.3390/molecules29194762

**Published:** 2024-10-08

**Authors:** Chen Tang, Yang Cao, Jie Gao, Gang Luo, Jiajun Fan, James H. Clark, Shicheng Zhang

**Affiliations:** 1 Department of Environmental Science and Engineering, Fudan University, Shanghai 200433, China; 21210740030@m.fudan.edu.cn (C.T.);; 2 Shanghai Key Laboratory of Atmospheric Particle Pollution and Prevention (LAP3), Shanghai Technical Service Platform for Pollution Control and Resource Utilization of Organic Wastes, Department of Environmental Science and Engineering, Fudan University, Shanghai 200438, China; 3 Shanghai Institute of Pollution Control and Ecological Security, Shanghai 200433, China; 4 Department of Civil and Environmental Engineering, The Hong Kong Polytechnic University, Hong Kong, China; 5 Department of Chemistry, Circa Renewable Chemistry Institute, Green Chemistry Center of Excellence, University of York, York YO10 5DD, UK

**Keywords:** lignin valorisation, aromatic compound, oxidative reaction, sustainable biorefinery

## Abstract

Lignin valorisation into chemicals and fuels is of great importance in addressing energy challenges and advancing biorefining in a sustainable manner. In this study, on the basis of the high microwave absorption performance of carbon nanotubes (CNTs), a series of copper-oxide-loaded CNT catalysts (CuO/CNT) were developed to facilitate the oxidative depolymerization of lignin under microwave heating. This catalyst can promote the activation of hydrogen peroxide and air, effectively generating a range of reactive oxygen species (ROS). Through the application of electron paramagnetic resonance techniques, these ROS generated under different oxidation conditions were detected to elucidate the oxidation mechanism. The results demonstrate that the ^•^OH and O_2_^•−^ play a crucial role in the formation of aldehyde and ketone products through the cleavage of lignin C_β_-O and C_α_-C_β_ bonds. We further evaluated the catalytic performance of the CuO/CNT catalysts with three typical lignin feedstocks to determine their applicability for lignin biorefinery. The bio-enzymatic lignin produced a 13.9% monophenol yield at 200 °C for 20 min under microwave heating, which was higher than the 7% yield via hydrothermal heating conversion. The selectivity of G-/H-/S-type products was slightly affected, while lignin substrate had a noticeable effect on the selective production. Overall, this study explored the structural characteristics of CuO/CNT catalysts and their implications for lignin conversion and offered an efficient oxidation approach that holds promise for sustainable biorefining practices.

## 1. Introduction

With the large consumption and limited amount of fossil resources, it is urgent that we seek renewable resources for sustainable development. At present, the utilization of lignocellulosic biomass from forestry, agriculture, and related industries can provide a possible route to obtain value-added chemicals and fuels from these bioresources [1,2]. Steps towards a renewable chemical industry can be achieved through carefully designed catalytic systems to enable cost-competitive and environmentally friendly processes [3,4]. Lignocellulosic biomass mainly includes cellulose, hemicellulose and lignin components. The conversion of cellulose and hemicellulose can produce platform chemicals and materials, which has been extensively studied over the years [5,6,7,8]. Lignin, consisting primarily of p-hydroxyphenyl (H), guaiacyl (G), and syringyl (S) units, is a rare example of a renewable source of aromatics, but the lack of efficient chemical conversion processes means that it is usually burned as fuel and not fully utilized. Converting lignin into high-value aromatic products can not only effectively alleviate the energy crisis, but also provide feasibility and commercial value for biorefinery [9,10].

The extraction and separation of lignin from lignocellulose will inevitably modify the natural structure of lignin. A variety of chemical and biological extraction methods have been explored for the production of kraft lignin, organosolv lignin, and enzymatic lignin [11,12,13]. It is widely recognized that lignin samples exhibit significant structural variability. Lignin has the characteristics of high molecular weight, complex structure, and easy repolymerization during the reaction process compared to simple model compounds, and usually requires harsh reaction conditions to increase the yield of aromatic compounds. The harsh treatment environment, such as kraft paper pulping by using the NaOH/Na_2_S process, will cause significant destruction of the lignin structure, so that the most abundant β-O-4 ether bond within the native lignin structure is cleaved [14]. Bio-enzymatic hydrolysis is a mild separation, and this process can retain most of the β-O-4 bonds in the lignin structure, which is conducive to obtaining a native lignin structure for further catalytic upgrading [15,16]. The organosolv extraction process is typically conducted in the low temperature range of 80–160 °C by using alcohols and organic acids, but this method may also inevitably break some lignin β-O-4 bonds. Catalytically converting lignin into more valuable compounds requires preserving its native structure, and in the process of lignin extraction, efforts should be made to prevent the destruction of the β-O-4 bond as much as possible. For sustainable biorefining, efficient systems capable of processing various types of lignin feedstocks must be developed.

Thermochemical, photochemical, and electrochemical conversion are the three major effective lignin conversion strategies [17,18,19,20,21]. Most research focuses on thermochemical reactions, mainly including oxidative/reductive depolymerization, high-temperature pyrolysis, and gasification [22,23,24,25,26]. Reductive depolymerization typically involves external H_2_ or hydrogen donor solvent (e.g., methanol, ethanol, and isopropanol) to convert lignin into aromatic hydrocarbons. However, this method faces challenges due to the high reaction temperature required and the necessity of external H_2_ [27,28]. In contrast, catalytic oxidative depolymerization provides a more favorable alternative, because this reaction can be conducted at milder reaction temperatures by using air, oxygen (O_2_), or hydrogen peroxide (H_2_O_2_) as oxidants [29,30]. Various valuable products, such as vanillin, acetovanillone, and syringaldehyde, can be produced from the oxidative depolymerization of lignin [31]. However, there are typically issues with lignin oxidative depolymerization, such as low monomer yields (5–20%), and the requirement for further high-pressure O_2_ addition (5–20 bar) and a high concentration of alkaline solution (1–4 mol/L of NaOH).

Extensive research has been conducted on the cleavage of C-O and C-C bonds in lignin model compounds, and efficient catalysts were designed to study the mechanism of lignin depolymerization and further improve the catalytic performance for natural lignin conversion and the yield of aromatic compounds. These catalysts can be broadly categorized into two groups: homogeneous catalysts, which include transition metal salts, oxometallates, ionic liquids [32,33,34], and heterogeneous catalysts, which involve active metal species loaded on various support such as carbons, Al_2_O_3_, and SiO_2_ [35,36]. Heterogeneous catalysts are easy to separate from the reaction system and can be recycled for long-term applications. Notably, copper oxides (CuO_x_) are often considered as active centers in multiple oxidation reactions. They exhibit higher redox ability and can generate an array of reactive oxygen species (ROS) by activating different oxidants (e.g., O_2_, air, and H_2_O_2_). These ROS, including hydroxyl radicals (^•^OH), superoxide anions (O_2_^•−^), and other highly reactive intermediates, have demonstrated the ability to selectively cleave the C-O and recalcitrant C-C bonds within the lignin structure. Therefore, developing environmentally friendly and low-cost Cu-based catalysts and understanding their key roles in practical lignin oxidation processes are crucial for future industrial applications.

Current research predominantly centers on the design of active sites to improve catalytic reactions. Few studies have explored the role of carbon support, particularly its advantages in microwave adsorption during reactions. Since microwave heating can quickly heat solid substrates without creating a temperature gradient, it has become an effective method for biorefineries [37,38,39,40,41]. In this approach, the temperature of the material is effectively raised by translating the alternating electromagnetic field into the thermal motion of dielectric molecules. With regard to a large specific surface area and exceptional thermal stability, carbon nanotubes (CNTs) have been employed as carbon-based catalysts [42,43,44]. Additionally, their larger dielectric loss coefficient indicates CNTs may have superior microwave absorption capacities, enabling the efficient conversion of microwave energy into thermal energy for thermochemical reactions [45]. Several studies have highlighted the potential of CNTs in enhancing the efficiency of advanced oxidation processes (AOPs) aimed at degrading organic pollutants when coupled with microwave-assisted reactions [46,47]. The enhanced degradation performance is attributed to the generation of more ROS on the catalyst surface. Concurrently, research has demonstrated successful lignin degradation through the utilization of various oxidation reagents [48]. Based upon these findings, we hypothesize that the synergistic utilization of CuO in conjunction with CNTs may enhance the availability of active sites and promote the generation of radical species due to the strong absorption of microwaves by CNT supports, which may enable the enhanced lignin conversion under microwave heating.

In this study, based on the high absorption of microwaves by CNTs, a series of CuO/CNT catalysts were prepared at varying calcination temperatures to regulate the active Cu species along with understanding their structural evolution. These heterogeneous catalysts were investigated for the microwave-assisted oxidation of lignin, employing H_2_O_2_ as an oxidant. To enhance the solubility of lignin and promote its conversion, a low-concentration NaOH solution (0.1–1.0 M) was used. Various reaction parameters (reaction time, temperature, catalyst, H_2_O_2_ dosage, and NaOH concentration) were thoroughly investigated to elucidate the critical factors that govern the conversion of lignin. A comparative analysis was also conducted to identify the impact of microwave heating reactions versus conventional hydrothermal reactions with the prepared catalysts. Additionally, three typical lignin feedstocks were selected evaluate the applicability of the proposed oxidation system and investigate the selective production of monophenols by selecting appropriate substrates. The detection of various ROS was conducted to gain a deeper understanding of their roles in the selective production through the cleavage of lignin C-O and C-C bonds, thus elucidating the mechanistic pathways involved in the oxidative depolymerization of lignin.

## 2. Result and Discussion

### 2.1. Characterization of CuO/CNT Catalysts

**Figure 1:**
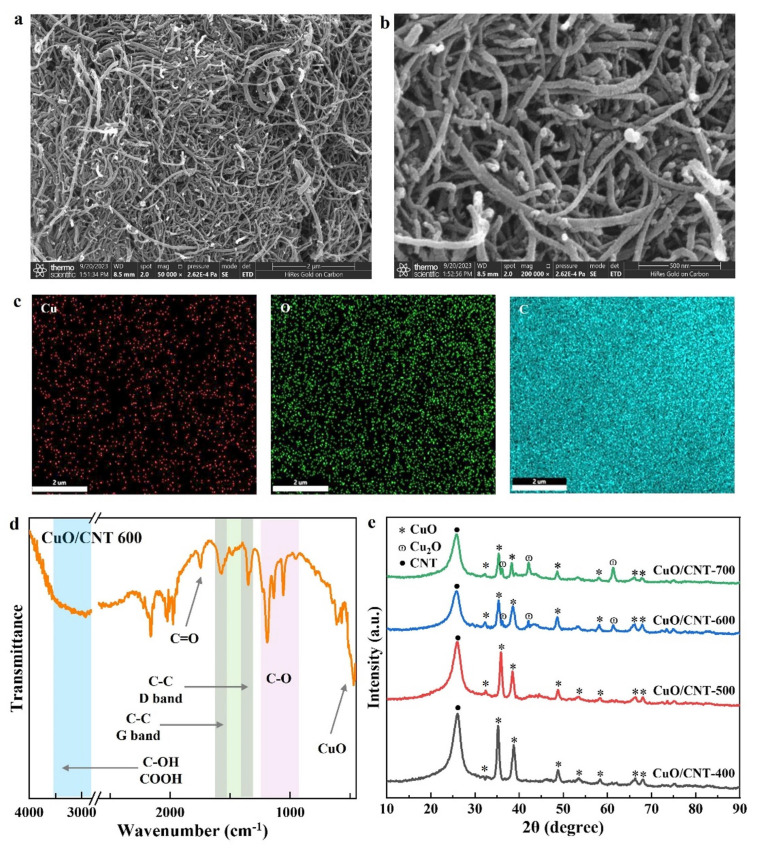
(**a**,**b**) SEM images of CuO/CNT 600 catalyst and (**c**) the element mapping of Cu, O, and C; (**d**) FTIR spectra of Cu/CNT 600; (**e**) XRD patterns of various catalysts.

The morphological characteristics of CuO/CNT catalysts were investigated by SEM analysis. As shown in Figure 1a,b, long and intertwined CNTs were observed without obvious agglomeration. These elongated CNTs played a crucial role in anchoring and dispersing CuO, thereby hindering its aggregation and preventing further growth on the supports [49]. Consequently, CuO species were found to be uniformly dispersed on the surface of the carbon nanotubes (Figure 1b). Additionally, SEM-EDX mapping confirmed the homogeneous dispersion of Cu, O, and C elements, thus providing more reactive sites for subsequent catalytic reactions (Figure 1c) [50]. In order to understand the surface functional groups of the as-prepared CuO/CNT catalyst, FTIR spectra were obtained (Figure 1d). Notably, the D and G bands at 1363 cm^−1^ and 1583 cm^−1^ were attributed to sp^3^ hybrid carbon defect and sp^2^ hybrid carbon material. The peaks at 1745 cm^−1^, 1153 cm^−1^, and 1073 cm^−1^ can be assigned to the stretching vibrations of C=O and C-O [51]. In addition, the surface abundance of carboxyl functional groups was also observed. These O-containing functional groups can enhance the hydrophilicity of the catalyst and promote its dispersion in water, which would also be of significant benefit for the liquid-phase reaction. The peak at 550 cm^−1^ attributed to Cu-O stretching vibration was observed, which provided evidence of the successful loading of CuO species onto the surface of CNT supports [52].

The phase and structure of the CuO/CNT catalysts synthesized at varying calcination temperatures were examined by XRD patterns. As shown in Figure 1e, the presence of a diffraction peak at 26° represents the carbon phase in CNT supports. Furthermore, the peaks at 35°, 38° and 48° confirmed the formation of CuO phases in all catalysts [53]. The crystal size of CuO of Cu/CNT 400 was estimated to be around 22 nm. At a higher calcination temperature of 600 °C, a discernible Cu_2_O diffraction peak is evident. With the increase in calcination temperature, the XRD peak intensity corresponding to CuO gradually weakens, while the peak intensity of Cu_2_O increased accordingly. This observation suggested that an excessively high calcination temperature may lead to a more significant carbothermal reduction in CuO to Cu_2_O by the carbon-based supports; the CuO/Cu_2_O exhibits superior catalytic performance for lignin depolymerization, which may be the reason why the yield of aromatic compounds increases with the increase in catalyst calcination temperature, thereby regulating the chemical state of the catalysts to further enhance the catalytic depolymerization of lignin [54].

The specific surface areas of the catalysts are summarized in Table 1. CuO/CNT 400 exhibited a high specific area of 222.8 m^2^/g, and its average pore diameter was 5.3 nm, representing a mesoporous structure. As the calcination temperature increased to 600 °C, the specific surface area and pore volume decreased slightly. The surface area of CuO/CNT 600 was maintained at 189.1 m^2^/g, and the pore volume was 0.23 cm^3^/g. However, the specific surface area of CuO/CNT 700 significantly decreased to 70 m^2^/g, which may be due to the aggregation of the CuO species blocking the pores of the support or the destruction of the CNT porous structure at higher temperatures, hindering the subsequent catalytic depolymerization of lignin and obtaining a lower product yield. For heterogeneous catalysis, creating a high specific surface area is important for enhancing catalytic activity. It provides more active sites to better come into contact with the lignin substrate, thus facilitating lignin conversion into aromatic products. Understanding the impact of calcination temperature on the textural structure of the catalysts provides valuable insights into the optimization of catalyst preparation.

**Figure 2:**
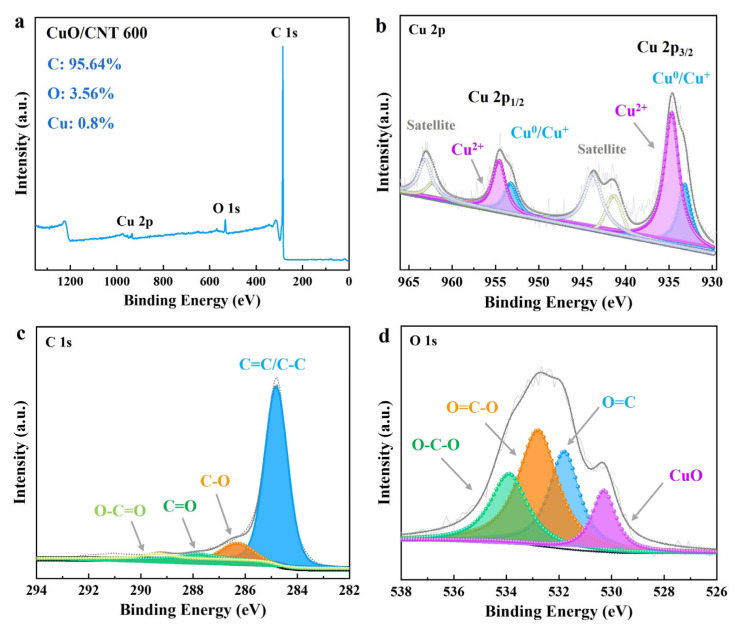
Chemical state of Cu catalysts. (**a**) The full spectrum scan of XPS spectra; (**b**) Cu 2p XPS spectra; (**c**) C 1s XPS spectra; (**d**) O 1s spectra for Cu/CNT 600.

XPS analysis further investigated the chemical state of the prepared catalysts. As shown in Figure 2a, CuO species were incorporated into carbon supports, as evidenced by the Cu peak in the XPS spectrum of CuO/CNT 600. Cu 2p_3/2_ XPS spectra showed two sets of peaks at binding energies of 933.1 eV and 934.6 eV that can be assigned to the Cu^+^/Cu^0^ and Cu^2+^ species, respectively, along with satellite peaks at 937–948 eV (Figure 2b). The binding energy at 954.1 eV was attributed to Cu 2p_1/2_, which also distinguished the Cu^+^/Cu^0^ and Cu^2+^ species in the catalyst [55]. In addition, the binding energies of 933.5 eV and 952.1 eV shifted to higher values compared to the pure CuO as the reference, indicating a strong metal–support interaction between CuO and CNT support [56,57] The enhanced interaction indicated a fast electron transfer ability on the catalyst surface, improving the overall catalytic efficiency during lignin conversion and enhancing hydrothermal stability. The C 1s XPS spectrum was deconvoluted into four sub-peaks. The peaks at 284.7, 286hen.3, 287.8, and 289.3 eV were assigned to C=C/C-C, C-O, C=O, and O-C=O species (Figure 2c). This result suggests that O-containing functional groups (i.e., carboxylic groups) are formed on the carbon support surface. We further analyzed surface oxygen species, as shown in Figure 2d. The O 1s peak of the catalyst was deconvoluted to four sub-peaks at the binding energies of 530.3, 531.8, 532.8, and 532.9 eV, corresponding to CuO, O=C, O=C-O, and O-C-O species, respectively. The results suggested that the predominant forms of surface oxygen are the carboxyl and carbonyl groups, which is consistent with the FTIR results.

### 2.2. Catalytic Performance of CuO/CNT for Lignin Depolymerization

**Figure 3:**
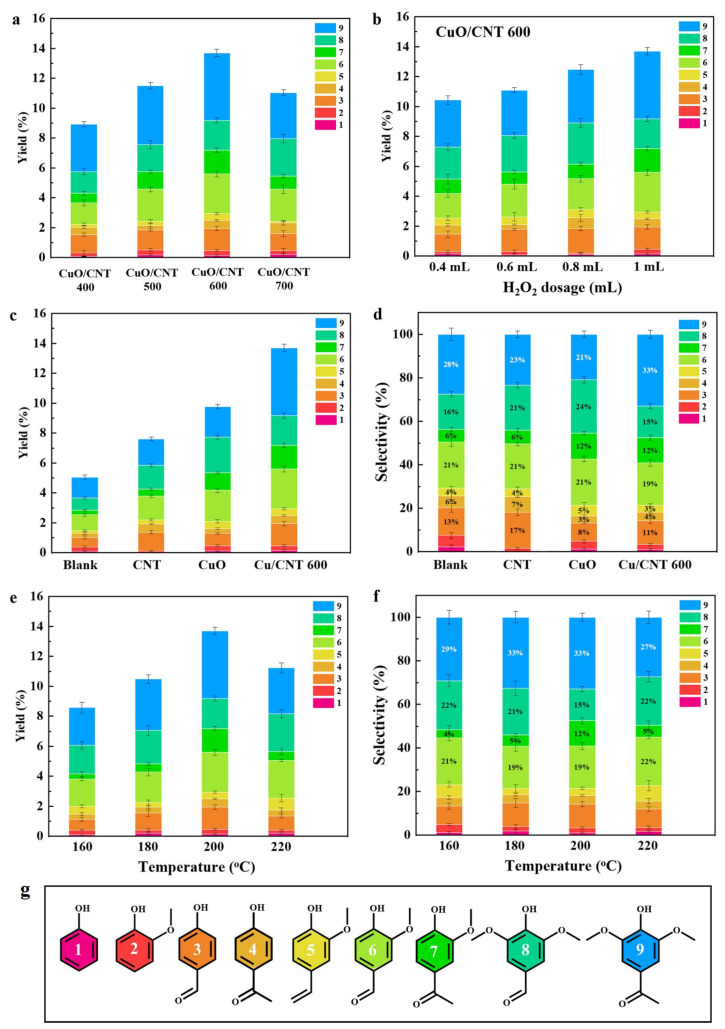
Effect of (**a**) catalyst calcination temperature and (**b**) H_2_O_2_ dosage on monophenol yield in the oxidative catalytic depolymerization of BL; (**c**) monophenol yield and (**d**) selectivity from BL depolymerization with various catalysts (200 °C, 20 min); effect of reaction temperature on (**e**) monophenol yield and (**f**) selectivity; (**g**) chemical structures of various monophenols.

Understanding catalyst properties/structures is crucial to elucidate the nature of active sites. In this regard, we found that the calcination temperature is a pivotal parameter with a profound impact on the chemical state of CuO_x_ species. The catalysts prepared at different calcination temperatures were investigated for lignin conversion. Figure 3a showed that the yield of monophenols exhibited an increased trend when using the catalysts prepared in a temperature range of 400 to 600 °C. However, the selectivity to monophenols was rarely affected. The chemical structures of the main products were shown in Figure 3g. This can be attributed to the high specific surface area and higher proportion of Cu_2_O in the catalysts prepared at high calcination temperatures. It has been reported that the mixed valence state of CuO and Cu_2_O exhibits superior performance in the oxidation process due to the enhanced redox ability. In this study, the more electron-rich Cu_2_O sites formed on the CNT surface were more favorable for lignin depolymerization. However, as the calcination temperature increased to 700 °C, a decrease in monophenol yield was observed. This decrease can be attributed to the aggregation of CuO species induced by higher calcination temperatures. Consequently, it significantly reduces the specific surface area of CuO/CNT catalysts from 189 m^2^/g to 70 m^2^/g, reducing the exposed surface to support active Cu sites for catalytic reactions [58]. These results indicated that the calcination temperature affects both the chemical state of CuO and morphologies of the catalysts, which further affect the subsequent lignin conversion. These findings provide valuable insights into the optimization of catalyst synthesis for monophenol production. H_2_O_2_ was used as an oxidant for lignin oxidation. Increasing the amount of H_2_O_2_ can generate more ROS to promote the oxidative depolymerization of lignin (Figure 3b), thereby increasing the yield of aromatics, but its effect on selectivity is minimal.

To identify the role of CuO and CNT in lignin depolymerization, the catalytic performance of various catalysts (e.g., CuO, CNT, and CuO/CNT) was evaluated in terms of monophenol yield and selectivity. As shown in Figure 3c,d, both CNT support and CuO enhance the production of monophenols in comparison to the blank controls. The selectivity to monophenols varied when using the CuO, CNT, and CuO/CNT catalysts. It seems that CuO promoted the production of product 8 and product 7, and CNT exhibited a higher selectivity towards product 3 and product 4. This distinction can be attributed to the differing oxidation pathways with carbon-based materials and CuO, and further EPR results have confirmed the different oxidation mechanisms. The highest monophenol yield of 13.9% was achieved with the CuO/CNT 600 catalyst at 200 °C for 20 min. The enhanced performance can be attributed to the fact that the abundant CuO-Cu_2_O active sites and the enhanced generation of ROS, as both the CuO and CNT can generate ROS, thus promoting lignin depolymerization into monophenols [47,59].

The reaction conditions were further investigated to understand the key reaction parameters influencing the selective production of monophenols. Figure 3e,f illustrated the variations in the yield and selectivity of monophenols as a function of reaction temperature. The results indicated a gradual increase in monophenol yield as the temperature is raised from 160 to 200 °C. However, this trend is observed to reverse as the temperature is further increased to 220 °C. The decreased monophenol yield at 220 °C is accompanied by a decrease in the yield and selectivity of syringone and vanillone products, while the yield and selectivity of syringaldehyde and vanillin products are increased. The results indicated that the oxidation of lignin to aldehydes was more favorable at higher reaction temperatures, while the S-/G-/H-type product distribution was rarely affected.

**Figure 4:**
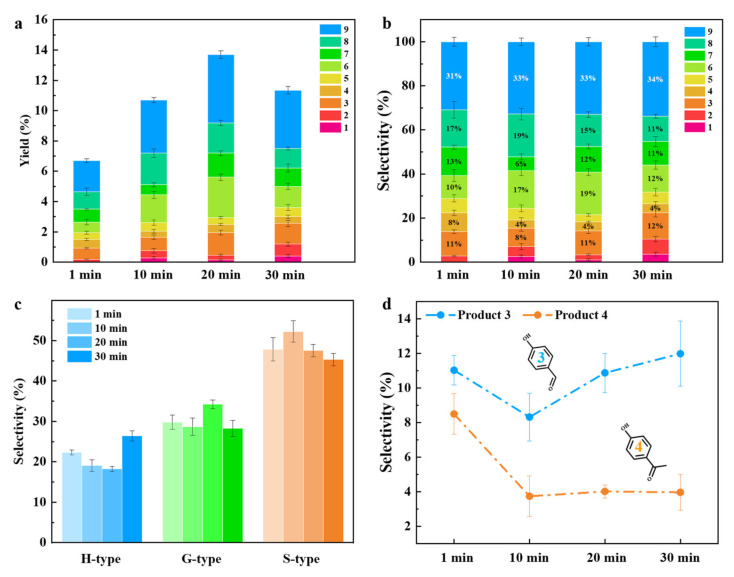
Effect of reaction temperature on (**a**) monophenol yield and (**b**) selectivity from BL depolymerization; selectivity of (**c**) H-/G-/S-type products and (**d**) P3 and P4 products at different reaction times. (Reaction conditions: 0.1 g BL, 0.15 g catalyst, 1 mL H_2_O_2_, 1 M NaOH, 200 °C).

Figure 4a showed a distinct trend in the yield of monophenol compounds at different reaction times. The yield gradually increased from 6.7% to 13.9% as the reaction time increased from 1 to 20 min. The cleavage of lignin β-O-4 bonds occurred relatively easily owing to its lower bond energy, mainly producing ketones and aldehydes [60]. When increasing the reaction time to 30 min, the produced aldehydes and ketones could be repolymerized/decomposed due to their poor thermal stability under alkaline conditions, consequently leading to a decrease in the overall yield (Figure 4b). Typically, the S-type and G-type products showed a higher thermal stability due to the -OCH_3_ group occupying specific positions within the benzene ring, impeding repolymerization reactions with other aromatic compounds. In addition, there is a significant decrease in H-type products at 10 min, which could be attributed to the repolymerization of product 3 and product 4 (Figure 4c,d). As the reaction time increased from 10 to 30 min, lignin was fully depolymerized, and product 3 was further generated, probably due to the defunctionalization of reactive products to H-type products, as products 8 and 9 were further generated into products 3 and 4 through C-O cleavage. The selectivity of product 4 remained relatively stable. This may be due to the fact that C-C in ketone compounds can promote the production of aldehyde products through reverse aldol condensation reaction in alkaline environments. Therefore, some products can be converted into product 3, and the lignin depolymerization, repolymerization, and defunctionalization of reactive products occurred during the oxidative processes.

**Figure 5:**
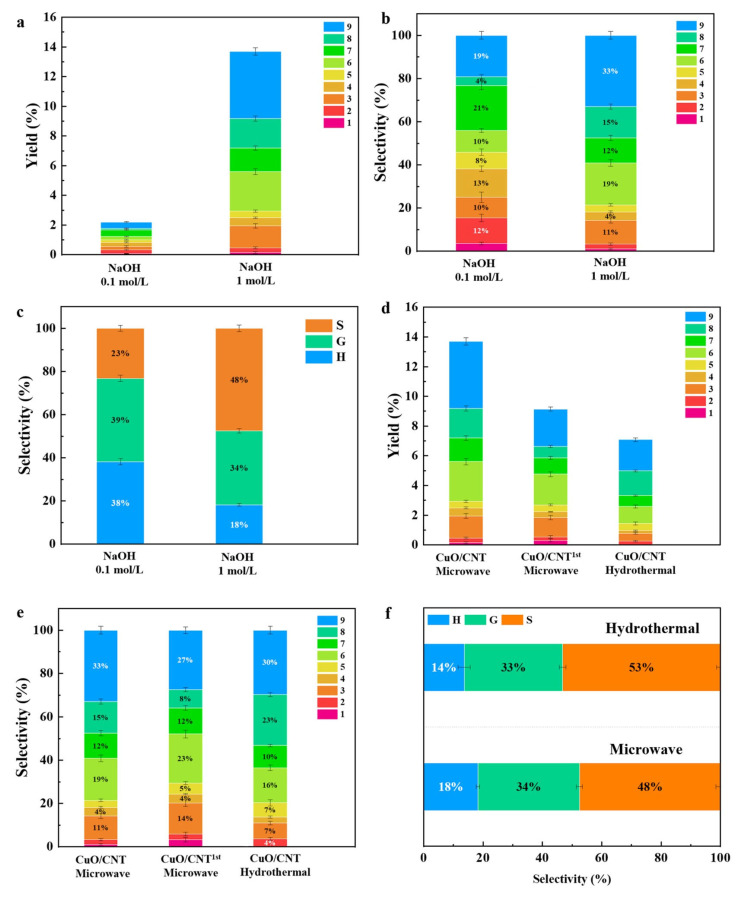
The effect of NaOH concentration on the (**a**) yield and (**b**) selectivity of monophenols; (**c**) selectivity of S-/G-/H-type products from BL depolymerization. Recyclability test on the (**d**) yield and (**e**) selectivity of monophenol yield from BL depolymerization with the CuO/BCN catalyst (microwave and hydrothermal); (**f**) selectivity of S/G/H-type products from BL depolymerization. (Reaction conditions: 0.2 g BL, 0.15 g catalyst, 1 mL H_2_O_2_, 1 M NaOH, 200 °C, 20 min).

**Figure 6:**
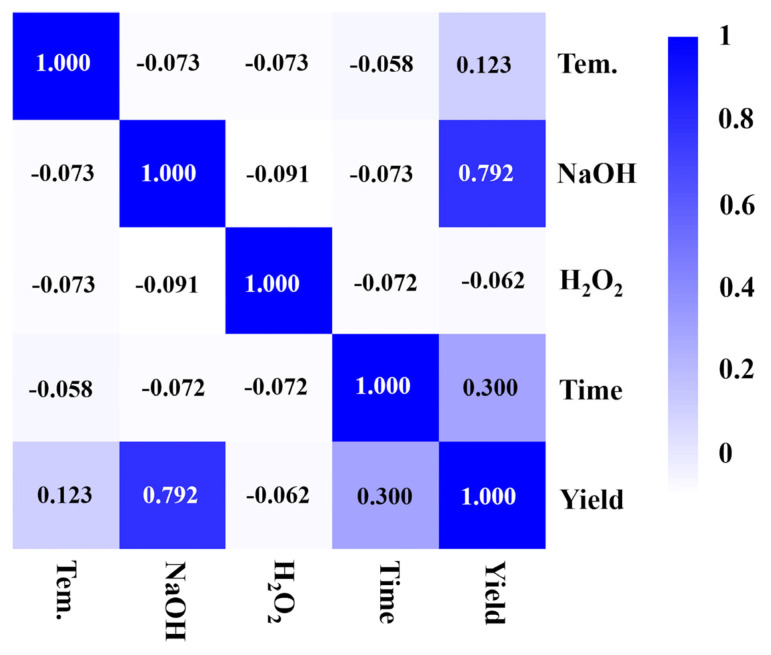
Pearson correlation map for reaction factors and monomer yields.

The influence of NaOH concentration on the yields of aromatic compounds has been investigated, as shown in Figure 5a. When the NaOH solution was at a concentration of 0.1 M, the yield of monophenols from the depolymerization of BL was remarkably low (2.2%). A significant improvement in the yield was observed upon increasing the NaOH concentration to 1 M. This improvement can be attributed to the effective lignin solubilization by NaOH, thereby facilitating lignin depolymerization during the liquid phase reactions. Furthermore, NaOH plays a pivotal role in generating aldehydes and ketones and significantly affects the selective production of more S-type products (48.2%) from BL, which could be due to the higher stabilities of these products (Figure 5b,c). The Pearson correlation coefficient (R), between several factors and conversion efficiency, can quantitatively elucidate the impacts of reaction parameters on lignin conversion to aromatics, as shown in Figure 6. A positive correlation between NaOH concentrations and yields was observed (R = 0.792), implying that the high concentration of NaOH is a key factor in enhancing monophenol production. These findings further demonstrate how specific reaction parameters impact the production of monophenols during lignin conversion, thereby offering valuable insights for further reaction parameter optimization.

**Figure 7:**
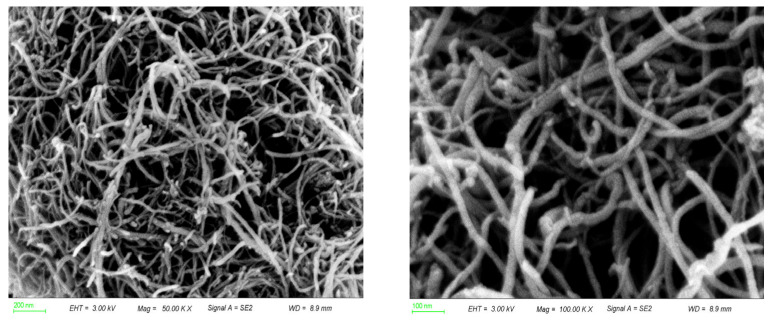
SEM images of the spent CuO/CNT catalysts after the reaction.

Microwave heating is considered a more environmentally friendly and sustainable method for lignin depolymerization [40]. The catalytic performance of CuO/CNT 600 under microwave heating and conventional hydrothermal liquefaction was compared. The results showed that microwave heating can increase the yield of monophenols compared with hydrothermal liquefaction, which indicates the superior efficacy of uniform microwave heating in facilitating lignin conversion into valuable phenolic compounds (Figure 5d). In addition, the prepared CuO/CNT catalysts may enhance the generation of ROS through the activation of H_2_O_2_ under microwave heating, as the CNTs have excellent microwave-absorbing properties. The impact of different reaction systems on the selectivity to H-/G-/S-type products was found to be significant (Figure 5e,f). In the microwave system, there was an increase in the production of H-type products, which may be attributed to the cleavage of the ester-linked *p*CA moiety. Conversely, in the hydrothermal systems with overheating, there was a higher yield of the more stable S-type product. After the first cycle, the product yield of the catalyst decreased from 13.9% to 9.2%, which may be due to the loss of some active components. However, compared with the blank and the water bath heating, the catalyst still has excellent catalytic performance (Figure 5d). Moreover, the recyclability test unveiled a slightly decreased yield. Notably, the morphology of the spent CuO/CNT catalysts remained after the cycle reaction (Figure 7). These results highlight the intricate interplay between different reaction conditions and the resultant product selectivity, offering valuable insights for optimizing catalytic processes in organic transformations.

### 2.3. Production of Monophenols from Different Lignin Substrates

To investigate the influence of lignin substrates on the monophenol production, three typical lignin feedstocks were selected for comparison. Table 2 shows the element analysis of different types of lignin and their molecular weights. All lignin samples showed high C contents ranging from 52.9 to 55.3%. BL exhibited a slightly higher O content attributable to oxygen-containing groups. A slightly increased sulfur content was observed in KL, probably due to the kraft extraction process involving the use of NaS [22]. The OL and BL obtained through mild extraction process showed lower molecular weights, which is conducive to depolymerization processes. Specifically, the molecular weight of BL is 925 Da, whereas the KL possessed a higher value of 10,000 Da, indicating a more significant degree of repolymerized structure in KL. The preservation of the natural lignin structure during extraction could contribute to its conversion into aromatic compounds.

**Figure 8:**
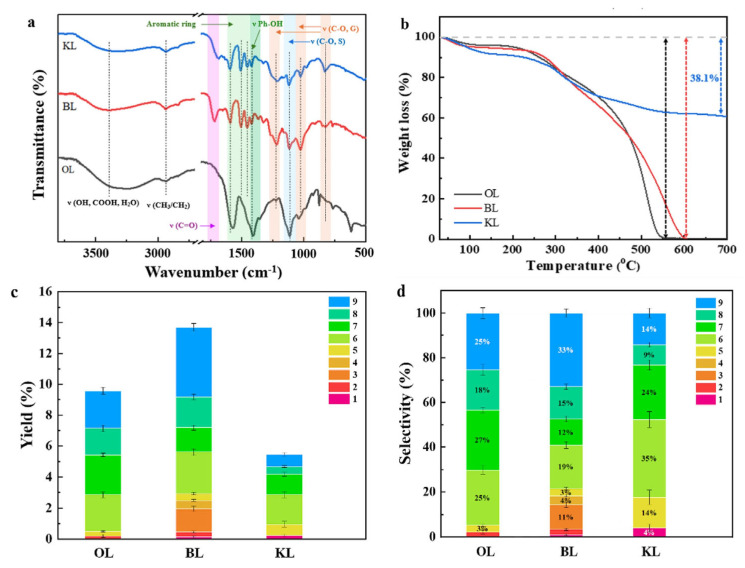
(**a**) FTIR spectra of lignin samples; (**b**) TG profiles of lignin samples; monophenol (**c**) yields and (**d**) selectivity with three types of lignin feedstocks. (Reaction conditions: 0.1 g lignin, 0.15 g catalyst, 1 mL H_2_O_2_, 1 M NaOH, 200 °C, 20 min).

The functional groups of the three lignin substrates were analyzed using FTIR. The 3343 cm^−1^ and 2937 cm^−1^ peaks can be attributed to the stretching vibrations of O-H and C-H in methyl and methylene groups, respectively (Figure 8a) [61]. Additionally, peaks at 1726 cm^−1^ for BL and 1692 cm^−1^ for OL were observed, corresponding to the stretching of C=O groups. Furthermore, the peaks observed at 1595 cm^−1^, 1507 cm^−1^, and 1422 cm^−1^ in various lignin samples were attributed to the stretching vibration of the aromatic ring C-C bonds, while the peak at 1460 cm^−1^ represents aromatic ring C-H stretching [62]. The peaks at 1215 cm^−1^ and 1035 cm^−1^ correspond to the stretching vibrations of the C-O and C-H bonds in the G-unit, respectively. Furthermore, the peaks observed at 1325 cm^−1^ and 1120 cm^−1^ represent the vibrations of the C-O and C-H bonds in the S-unit [63]. The FTIR results illustrated that the OL possessed abundant S-type units. while the KL mainly contains G-type units.

The thermochemical properties of different types of lignin were analyzed by TG. Both OL and BL show a complete weight loss at 600 °C under air conditions, indicating a high susceptibility to decomposition for these lignin feedstocks (Figure 8b). In contrast, KL retains 63% of its weight even at a higher temperature of 700 °C. This difference in thermal stability can be attributed to the structural alterations that occur in KL during this extraction process. The severe extraction conditions lead to the significant degradation of the KL structure, forming more stable C-C bonds through repolymerization, as reported by our previous studies [64]. The condensed structure of KL indicates that a harsh reaction condition may be required to break C-C bonds to produce monophenols [65].

The investigation into the yields of monophenols derived from three distinct lignin substrates is shown in Figure 8c,d and Supplementary Materials Table S1. It was observed that the yields of aromatics from the depolymerization of BL and OL were higher, which could be due to their cleavable structures, as indicated by their lower decomposition temperatures in TG profiles. Additionally, different lignin substrates illustrated varying product selectivity in the oxidative reactions. The main products from OL and BL were S-type and G-type aldehydes and ketones, which were mainly formed by the cleavage of C-O and C-C bonds in the S-type and G-type structures, and the H-type products in BL may be obtained through the cleavage of C-C and C-O in G-type structures, which is consistent with the FTIR conclusion that BL contains more G-type structures, while OL contains more S-type structures. This is consistent with the FTIR conclusion that BL lignin contains more G-type structures, while OL contains more S-type structures. Meanwhile, higher S/G-type structures in OL and BL were evidenced by FITR spectra, which can result in the selective production of these monophenols and makes it easier to obtain high yields [66]. A high selectivity to stable G/S-type products and a low yield were observed with KL substrate. This can be attributed to the condensed structure of KL that is formed during the harsh extraction process. The complex structure of KL makes it more resistant to degradation, as evidenced by the TG results, resulting in a lower yield of desired products. In addition, the low yield of S-type products can be attributed to their natural structure having fewer S-units, which is consistent with the FTIR results.

### 2.4. Mechanism Study

**Figure 9:**
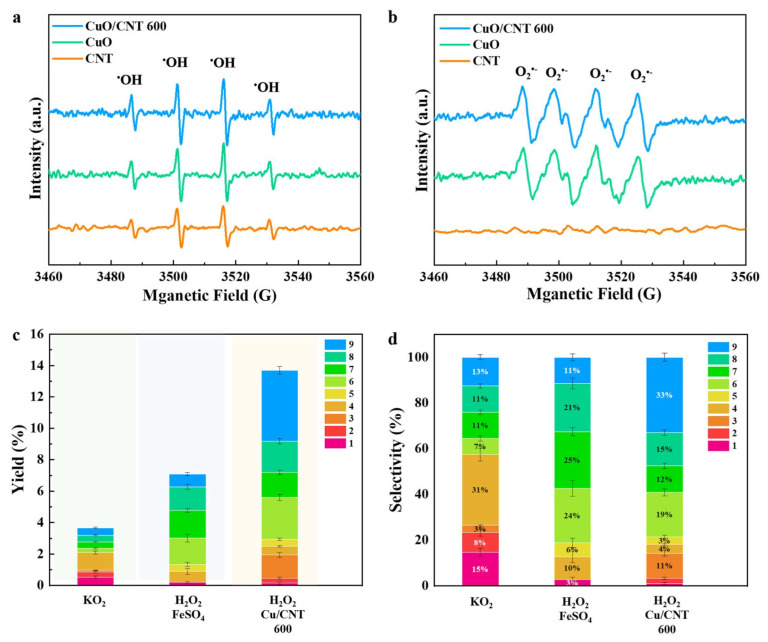
EPR detection of (**a**) hydroxyl radicals and (**b**) superoxide radicals; (**c**) monophenol yield with different radicals involved reactions; (**d**) selectivity of the products with different radicals involved reactions. (Reaction conditions: 0.1 g BL, 0.15 g catalyst, 1 M NaOH, 200 °C, 20 min).

To study the reaction mechanism of oxidative depolymerization of lignin, we used EPR to identify the ROS involved in the reactions. Figure 9a showed distinct signal peaks corresponding to hydroxyl radicals when CuO, CNT, and CuO/CNT were introduced into the reactions. All Cu-based catalysts can generate ^•^OH radicals through the activation of H_2_O_2_, while CNT may absorb microwave energy to promote the formation of ^•^OH radicals. It is noteworthy that the CuO/CNT catalyst exhibits the highest intensity of ^•^OH diffraction peak, indicating the potential synergistic effect of CuO and CNT in the generation of ^•^OH radicals.

**Figure 10:**
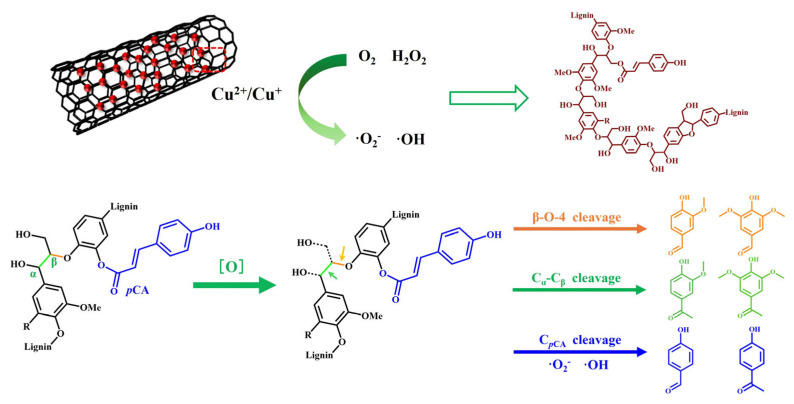
Proposed oxidative pathway for lignin depolymerization by ROS.

Furthermore, the six characteristic peaks of the DMPO-O_2_^•−^ radical sites can be observed with both the CuO and CuO/CNT catalysts (Figure 9b). The absence of diffraction peaks in the CNT system suggested that O_2_^•−^ radicals generated by CuO/CNT may originate from copper oxides. Additional experiments were conducted to elucidate the role of these radicals in lignin depolymerization and the production of resultant products. Specifically, a Fenton reaction (H_2_O_2_ and FeSO_4_) was employed to generate ^•^OH radicals, while potassium peroxide (KO_2_) was utilized to only produce O_2_^•−^ radicals [67]. In the O_2_^•−^ radical system alone, the yield of monophenols is low (3.5%), mainly obtaining H-type products. The results indicated that the O_2_^•−^ radicals may contribute to the cleavage of ester-linked *p*CA moiety and remove the side chains of the monophenols. When the H_2_O_2_ system with FeSO_4_ was explored, ^•^OH radicals were generated and mainly contributed to the cleavage of C-O and C_α_-C_β_, resulting in a 6.8% yield of monophenol with a high selectivity to vanillin (G-type product), acetovanillone (G-type product) and syringaldehyde (S-type product) (Figure 9c,d). From the overall yield of aromatic compounds, hydroxyl radicals have higher activity in lignin depolymerization and generate more aromatic monomers. In H_2_O_2_ system with CuO/CNT, both H_2_O_2_ and air can be activated to generate ^•^OH and O_2_^•−^, as evidenced by EPR results. The highest yield of monophenol was achieved through the cleavage of the C_α_-C_β_ linkage, yielding vanillin (product 6) and syringaldehyde (product 8), as well as the cleavage of the β-O-4 bond, producing vanillone (product 7) and syringone (product 9), indicating both ^•^OH and O_2_^•−^ radicals were involved in the oxidative depolymerization of lignin (Figure 10).

## 3. Methods

### 3.1. Lignin Samples

The bio-enzymatic lignin (BL isolated from corn cob) was purchased from Shandong Longli Biotechnology. In the process of extracting organosolv lignin (OL) from cottonwood, a mixture of formic acid, acetic acid, and water was utilized. The wood biomass was thoroughly stirred with the solvent mixture and heated in an oil bath at 105 °C for 8 h. The resulting mixture was filtered to separate the residue from the filtrate. Then, a large amount of ice water was added to the filtrate, and the OL was collected as a precipitate. This method proved effective in isolating lignin from the wood biomass. Kraft lignin (KL) was purchased from Sigma Aldrich (Shanghai, China) Trading Co., Ltd. [64].

### 3.2. Preparation of CuO/CNT Catalyst

For the preparation of CuO/CNT, 0.5 g CNT was added to a solution comprising ethanol and water in a 1:1 ratio, followed by thorough stirring to ensure uniform distribution. Then, 0.2 g Cu(NO_3_)_2_·3H_2_O was added to the solution, which was then subjected to ultrasound treatment for 10 min. Magnetic stirring was then carried out at 120 °C, and the mixture was boiled until the solution volatilized to a sticky state. The colloid was dried overnight at 60 °C and then heated to 600 °C for 3 h at a heating rate of 2 °C/min in an air atmosphere to obtain CuO/CNT 600 catalysts with a CuO loading of 5%. The catalysts calcined at different temperatures (400, 500, 600, and 700 °C) were prepared for comparison. All catalysts were sieved through a 100-mesh screen.

### 3.3. Microwave-Assisted Oxidative Depolymerization of Lignin

In a typical run, 0.2 g of lignin was dissolved in 10 mL of 1 M NaOH solution through ultrasound. Then, 0.15 g of catalyst and 1 mL of H_2_O_2_ (30 wt%) were introduced into the reactor before heating. The utilization of microwave-assisted heating facilitated the decomposition of H_2_O_2_, thereby creating an oxidative environment conducive to lignin depolymerization. To enhance catalytic efficiency, four temperature gradients were established at 160, 180, 200, and 220 °C. Additionally, 0.15 g CNT and 0.15 g CuO were used for control experiments. The reactor was heated to the desired temperature within 7 min using 1000 W power. After the reaction, the microwave reactors were quickly cooled to room temperature by air cooling. The solid catalyst was collected by filtration and then washed three times with deionized water and ethanol and dried at 60 °C. Subsequently, the reaction solution pH was adjusted to 2 by using diluted HCl, and the liquid products were extracted by ethyl acetate for further analysis.

### 3.4. Qualitative and Quantitative Analysis of Products

The products obtained from the oxidative depolymerization of lignin were qualitatively and quantitatively analyzed by gas chromatography–mass spectrometry (GC-MS) (Agilent GC7890N and matching Wax column, Santa Clara, CA, USA). Dodecane was used as the internal standard to quantify the yield of monophenols [68].

$$Yield\;\left(\%\right)=\frac{m_{product}^{final}}{m_{lignin}^{initial}}\times 100\%$$

$$Selectivity\;\left(\%\right)=\frac{m_{product}^{final}}{\sum m}\times 100\%$$

### 3.5. Characterization of Catalyst and Lignin

Rigaku Ultima IV multifunctional X-ray diffraction (XRD) was used to analyze the crystal structure of the catalyst (λ = 0.15418 nm, 40 kV, 40 mA). Scanning electron microscopy (SEM, SU8010, Hitachi, Tokyo, Japan) and SEM with energy dispersive X-ray spectroscopy (SEM-EDX) were used to analyze the surface structure and element distribution of the catalysts, and the acceleration voltage was from 0.2 kV to 30 kV. X-ray photoelectron spectroscopy (XPS, EMAX50, Tokyo, Japan) was used to analyze the surface element composition and chemical valence of the catalyst. The chemical structure and functional group of the catalyst were analyzed by Fourier transform infrared spectroscopy (FTIR, Nicolet IS5, Madison, WI, USA). The molecular weight distribution of three different types of lignin was determined using a gel permeation chromatography (GPC) instrument (Agilent 1260HPLC Systems, Santa Clara, CA, USA) equipped with a refractive index detector (RID); the mobile phase is tetrahydrofuran, and the flow rate is 1mL/min. Element analysis (Elementar Vario EL III, Frankfurt, Germany) was used to determine the contents of C, H, O, and S in lignin samples. Thermogravimetric analysis (TG, PerkinElmer TGA 8000, Waltham, MA, USA) was used to quantitatively measure the change in mass and the change rate of mass. The lignin samples were placed in a porcelain crucible and then heated up to 700 °C with a heating rate of 20 °C/min under an air atmosphere. The electron paramagnetic resonance (EPR) spectra were obtained using A300 spectrometer (A300, Bruker, Bremen, Germany) to detect ROS. In the sample vials, 100 mM 5,5-dimethyl-1-pyrroline-N-oxide (DMPO) was added to the water or methanol solution. The peak intensities of DMPO-OH, DMPO-O_2_^•–^, and 2,2,6,6-tetramethyl-4-piperidinol (TEMP)-^1^O_2_ signals at 20 min were applied as indexes of the generated ROS after the correction of background noise.

## 4. Conclusions

In this work, on the basis of the high microwave absorption performance of CNTs, we developed CuO/CNT catalysts that enable the activation of H_2_O_2_ and air for lignin depolymerization under microwave heating. The calcination temperature plays a pivotal role in the speciation of CuO_x_ and catalyst structural properties. To evaluate the catalytic performance, the yields and selectivity of monophenols were assessed for CuO/CNT catalysts prepared at varying calcination temperatures (400–700 °C). The results demonstrated that the CuO/CNT 600 catalyst exhibits a higher specific surface area and demonstrated the co-existence of CuO-Cu_2_O species, which could facilitate the conversion of lignin into valuable monophenols. Through a systematic optimization of several reaction parameters, we investigated the key factors determining the yield of monophenols. A notable 13.9% yield was achieved at 200 °C for 20 min when employing BL as the feedstock. This study was further expanded to encompass the oxidative depolymerization of three different types of lignin to diversify the array of aromatic compounds obtained. EPR experiments verified the pivotal roles of ^•^OH and O_2_^•−^ radicals in the cleavage of lignin C-O and C-C bonds, ultimately leading to distinct depolymerization pathways and variations in the selective production of monophenols.

## Figures and Tables

**Table 1:** Textural structure of various catalysts.

Catalyst	S_BET_ (m^2^/g)	V ^a^ (cm^3^/g)	D ^b^ (nm)
CuO/CNT 400	222.8	0.29	5.3
CuO/CNT 500	201.5	0.19	2.4
CuO/CNT 600	189.1	0.23	4.8
CuO/CNT 700	70.0	0.22	3.6

^a^ Total pore volume of pores; ^b^ Average of pores width.

**Table 2:** Element analysis of various types of lignin (wt%).

Lignin	N	C	H	S	O	M_w_ (Da)
OL	0.14	55.34	5.72	0	38.8	1950
BL	0.77	52.92	5.54	0	40.77	925
KL	0.11	53.77	5.28	2.88	37.96	10,000

## Data Availability

The original contributions presented in the study are included in the article and Supplementary Materials.

## Supplementary Materials

The following supporting information can be downloaded at: https://www.mdpi.com/article/10.3390/molecules29194762/s1, Table S1. Yield and selectivity of S, G, and H under different conditions.
