# Supplementary material for: Oxidative Catalytic Depolymerization of Lignin into Value-Added Monophenols by Carbon Nanotube-Supported Cu-Based Catalysts
Source: Molecules. 2024 Oct 8;29(19):4762. doi: 10.3390/molecules29194762 (PMC11478243; doi:10.3390/molecules29194762)
Supplement: Supplementary file 1 [file molecules-29-04762-s001.zip › molecules-3225412-supplementary.pdf]

Table S1. Yield and selectivity of S, G, and H under different conditions

Reaction conditions	S-Products		G-Products		H-Products	
	Yield	Selectivity(%)	Yield	Selectivity(%)	Yield	Selectivity(%)
CuO/CNT 400	4.63	51.85	2.27	25.42	2.03	22.73
CuO/CNT 500	5.76	50.04	3.59	31.20	2.16	18.76
CuO/CNT 600	6.51	47.51	4.69	34.23	2.50	18.26
CuO/CNT 700	5.60	50.68	3.13	28.32	2.32	21.00
0.4mL H_2O_2	5.28	50.62	3.07	29.43	2.08	19.95
0.6mL H_2O_2	5.47	49.27	3.52	31.71	2.11	19.02
0.8mL H_2O_2	6.33	50.76	3.58	28.70	2.56	20.52
1.0mL H_2O_2	6.51	47.51	4.69	34.23	2.50	18.26
Blank	2.21	43.77	1.54	30.49	1.30	25.74
CuO	4.44	45.40	3.73	38.14	1.61	16.46
CNT	3.36	44.09	2.33	30.59	1.93	25.32
160℃	4.42	51.57	2.67	31.15	1.48	17.28
180℃	5.66	54.00	2.87	27.38	1.95	18.62
200℃	6.51	47.51	4.69	34.23	2.50	18.26
220℃	5.57	49.60	3.91	34.82	1.75	15.58
OL	4.16	43.42	5.21	54.39	0.21	2.19
BL	6.51	47.51	4.69	34.23	2.50	18.26
KL	1.27	23.30	3.96	72.66	0.22	4.40
